# Mutations in a plastid-localized elongation factor G alter early stages of plastid development in *Arabidopsis thaliana*

**DOI:** 10.1186/1471-2229-7-37

**Published:** 2007-07-13

**Authors:** Nicholas J Ruppel, Roger P Hangarter

**Affiliations:** 1 Department of Biology, Indiana University, Bloomington, IN, 47405, USA

## Abstract

**Background:**

Proper development of plastids in embryo and seedling tissues is critical for plant development. During germination, plastids develop to perform many critical functions that are necessary to establish the seedling for further growth. A growing body of work has demonstrated that components of the plastid transcription and translation machinery must be present and functional to establish the organelle upon germination.

**Results:**

We have identified *Arabidopsis thaliana *mutants in a gene that encodes a plastid-targeted elongation factor G (*SCO1*) that is essential for plastid development during embryogenesis since two T-DNA insertion mutations in the coding sequence (*sco1-2 *and *sco1-3*) result in an embryo-lethal phenotype. In addition, a point mutation allele (*sco1-1*) and an allele with a T-DNA insertion in the promoter (*sco1-4*) of *SCO1 *display conditional seedling-lethal phenotypes. Seedlings of these alleles exhibit cotyledon and hypocotyl albinism due to improper chloroplast development, and normally die shortly after germination. However, when germinated on media supplemented with sucrose, the mutant plants can produce photosynthetically-active green leaves from the apical meristem.

**Conclusion:**

The developmental stage-specific phenotype of the conditional-lethal *sco1 *alleles reveals differences in chloroplast formation during seedling germination compared to chloroplast differentiation in cells derived from the shoot apical meristem. Our identification of embryo-lethal mutant alleles in the Arabidopsis elongation factor G indicates that *SCO1 *is essential for plant growth, consistent with its predicted role in chloroplast protein translation.

## Background

In oilseed plants such as Arabidopsis (*Arabidopsis thaliana*) and rapeseed (*Brassica napus*), developing embryos are green and cells in these embryos develop functional chloroplasts [1]. The green embryos are capable of photosynthesis and have been shown to fix carbon crucial to the biosynthesis of seed storage oils [2-4]. In experiments with cultured rapeseed embryos and siliques, light was found to increase embryo growth-rates, which correlated both with improved carbon sequestration and with its utilization in seed oil synthesis [5]. These effects were largely negated by inhibition of photosynthesis, and their studies indicated that it is the reductant and/or ATP produced by photosynthesis in green embryos that is important for normal embryo growth and seed development.

Arabidopsis embryos begin to develop chloroplasts and appear green around 5 days after pollination and the chloroplasts remain present for up to approximately 12 days after pollination [6]. When seeds are maturing during late embryogenesis (>12 days), the chloroplasts dedifferentiate and lose their chlorophyll, starch, and internal membranes to seed storage reserves, which results in the formation of white embryos in mature Arabidopsis seed [6]. In soybean (*Glycine max*), a small simple plastid called an eoplast has been found in fully mature embryo cells [7]. Eoplasts resemble proplastids but are derived from chloroplasts. Thus far, eoplasts have not been observed in mature Arabidopsis embryos, largely because the embryo cells are so densely packed with lipid and protein bodies [8]. Nevertheless, a basal-state plastid must be maintained in the cells of fully mature Arabidopsis embryos since chloroplasts, amyloplasts, and the various other plastid types re-develop upon seedling germination. The development of these plastids early after germination can be critical for seedling survival since, in addition to photosynthesis and the production of starch, plastids are also involved in the biosynthesis of fatty acids [9], nucleic acids, and amino acids [10].

Although a good deal is known about the physiological and biochemical functions of chloroplasts during embryo growth and seed production, investigations of chloroplast development during embryogenesis have been largely descriptive [1,11]. To identify molecular components involved in plastid development during the youngest phase of the Arabidopsis life cycle, a screen was conducted to identify mutants that specifically influence plastid development in embryos and seedling tissues derived from the embryo, but not in tissues derived from the apical meristem. Mutants were identified that exhibited cotyledon and hypocotyl albinism upon germination due to improper chloroplast development, while photosynthetic tissues derived from the shoot apical meristem were green and appeared to develop normal chloroplasts. This paper describes mutants in a gene that encodes for a plastid-localized elongation factor G (EF-G). One mutant allele from an EMS-(ethyl methanesulfonate) mutagenized population was found to be the result of the same nucleotide substitution responsible for the recently described *s*nowy *co*tyledon *1 *(*sco1*) [GenBank:NM_104952] mutant [12]. Two alleles with T-DNA insertions directly in this gene resulted in embryo lethality, demonstrating that this EF-G is essential during embryogenesis. Our analysis of the different mutant alleles of *SCO1 *indicate that differentiation of eoplasts to chloroplasts during germination may have different requirements for protein translation than for proplastid to chloroplast differentiation in cells derived from the apical meristem.

## Results

### Embryo-lethal *sco1 *alleles

**Figure 1:**
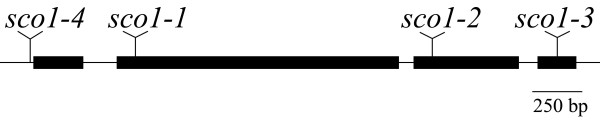
**The *SCO1 *mutational map**. *SCO1 *encodes for a predicted protein with a high degree of similarity to an EF-G containing a chloroplast localization signal (At1g62750). The locations of the 4 mutant alleles are indicated. The EMS-mutagenized *sco1-1 *allele represents a G to A base change within the GTP-binding domain of the gene, which converts a glycine at amino acid 132 to an arginine. The other alleles [*sco1-2 *(Salk_046154), *sco1-3 *(Salk_039084), and *sco1-4 *(Salk_025112)] were isolated from the Salk T-DNA insert collection.

**Figure 2:**
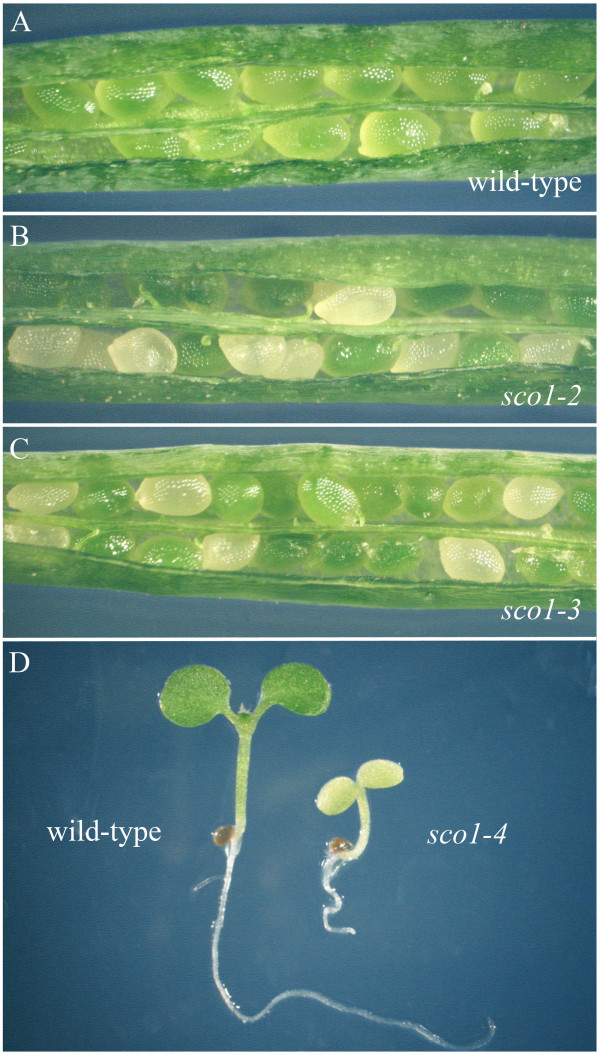
**Phenotypes of T-DNA insertion alleles**. In 9-d-old siliques, wild-type Arabidopsis ovules were green (A), but in siliques from *sco1-2 *(B) and *sco1-3 *(C) heterozygotes, white ovules were found intermixed with normal green ovules, indicating that chloroplast development is disrupted during embryogenesis in these alleles. White ovules accounted for approximately 25% of the total observed (*sco1-2*, 88 white ovules out of 360; *sco1-3*, 36 white ovules in 166). The upstream T-DNA insertion in 5-d-old *sco1-4 *seedlings (D, right) resulted in significantly stunted growth and pale cotyledons when compared to wild-type seedlings of similar age (D, left).

Two alleles of *sco1 *(*sco1-2 *and *sco1-3*) isolated from the Salk T-DNA insert collection have T-DNA inserts at the C-terminal end of the gene (Figure 1). In *sco1-2*, all of the T4 seed tested developed green cotyledons. Genotypic analysis of the viable progeny showed that these plants consisted of a segregating population with a ratio of 1:2 for wild-type to heterozygote for the T-DNA insert in the *SCO1 *gene. No viable progeny were found that were homozygous for the T-DNA insert. Moreover, examination of developing siliques on plants that were heterozygous for the *sco1-2 *T-DNA insert revealed that approximately 25% of the developing ovules were white (88 white ovules out of 360 examined; see Figure 2B). The 9 d-old white ovules were similar in size to the green ovules, but upon dissection they did not appear to contain a developing embryo. This was presumably due to abortion of the embryo at a very early stage of embryogenesis. Similar results were observed for the *sco1-3 *T-DNA insertion line (Figure 2C). These findings indicate that null mutants in *SCO1 *are lethal early during embryo development and that the EF-G encoded by *SCO1 *is essential for plant development.

### Conditional seedling-lethal *sco1 *alleles

**Figure 3:**
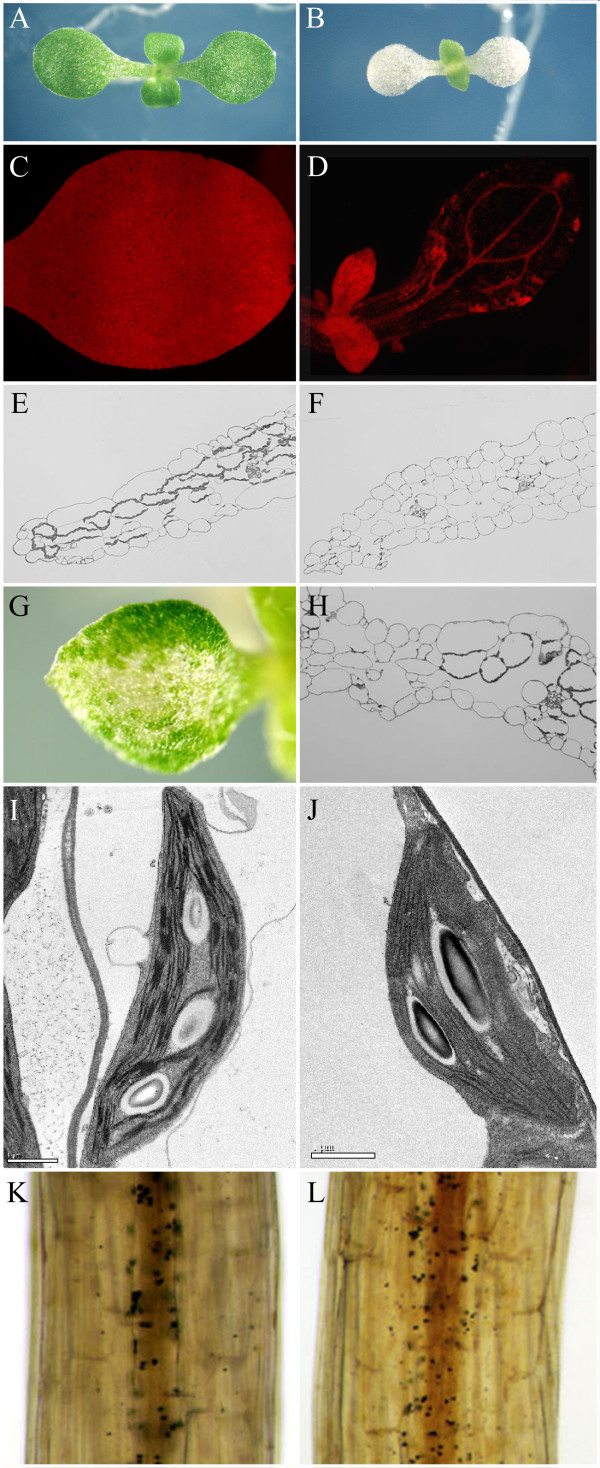
**Characterization of the *sco1-1 *mutant**. A-J are from 5-d-old light-grown seedlings, while K and L are from 4-d-old dark-grown seedlings. Upon seedling germination in white-light, the cotyledons of *sco1-1 *(B) appear colorless compared to wild type (A), but leaves that emerge from the apical meristem are green like wild-type leaves. Chlorophyll autofluorescence and cotyledon cross sections show that *sco1-1 *cotyledon cells (D and F) are almost completely devoid of chloroplasts except in cells associated with the vasculature, while wild-type (C and E) show a normal complement of chloroplasts in cotyledon cells. The albinism phenotype of *sco1-1 *is not always complete and green cells can be found in some *sco1-1 *cotyledons, where they are typically located along the margins of the tissue (G). A cross section of a cotyledon (H) from such a variegated mutant shows cells with a normal complement of chloroplasts adjacent to cells devoid of chloroplasts. Ultrastructural analysis of chloroplasts in these 'sectored' *sco1-1 *cotyledon mesophyll cells (J) showed that they are similar to chloroplasts in wild-type cotyledons (I). Starch deposition in 4 d-old dark-grown wild-type (K) and *sco1-1 *hypocotyls (L) appears similar, indicating that amyloplast development is not severely affected in the *sco1-1 *mutant. Scale bars in I and J are 1 μm.

We identified one EMS-derived *sco *mutant that, upon mapping and sequencing, was found to be identical to the *sco1 *allele recently identified by Albrecht et al. [12], where a G to A base change converted a conserved glycine residue to an arginine. We have designated this allele as *sco1-1 *(Figure 3B). Seedlings of *sco1-1 *rarely survived past the cotyledon stage unless they were provided with supplementary carbon (Table 1). In addition, an allele (*sco1-4*) with a Salk T-DNA insert located 14 base-pairs upstream of the *SCO1 *ATG start site was found to have a similar seedling-lethal phenotype. However, *sco1-4 *plants could also be rescued when germinated on medium supplemented with sugar (Table 1). Unlike the white cotyledons of *sco1-1 *(Figure 3B), *sco1-4 *seedlings had very pale green cotyledons (Figure 2D). When germinated on media with sucrose, the first true leaves that emerged from *sco1-4 *seedlings were initially pale, but when transplanted to soil, the seedlings were able to survive and the rosette leaves of adult plants resembled wild type (data not shown).

**Table 1:** Effect of sucrose on survival of wild-type and *sco *mutants. On 0.5-strength MS media supplemented with 2% sucrose, wild-type, *sco1-1*, and *sco1-4 *seedlings have comparable survival rates. In the absence of sucrose, less than 20% of the *sco1-1 *seedlings were able to produce true leaves and survive into adulthood, and no *sco1-4 *seedlings survived. Typically, the *sco1-1 *mutant seedlings that survived to adulthood in the absence of a supplemental carbon source were ones that had larger patches of green cells in their cotyledons and hypocotyls

Genotype	+ Sucrose	- Sucrose
Col-0	95/98 = 97%	84/94 = 89%
*sco1-1*	84/92 = 91%	17/89 = 19%
*sco1-4*	25/26 = 96%	0/21 = 0%

### Plastid development in the *sco1 *mutant

Although most *sco1-1 *cotyledons appear completely white, fluorescence microscopy revealed some red autofluorescence is present in all mutant cotyledons, especially along the vasculature (Figure 3D). The red autofluorescence, which is indicative of the presence of chlorophyll, suggested that chloroplast development was not completely blocked in the mutant seedlings despite the visually albino appearance. Also, upon closer examination of some *sco1-1 *seedlings, we often observed patches of green cells, which were typically located near the margin of the cotyledons and in the upper hypocotyl (Figure 3G). The extent of this sectoring phenotype varied from seedling to seedling. In the absence of supplemental sugar, over 80% of the *sco1-1 *seedlings failed to produce true leaves and died (Table 1). The mutant seedlings that were able to survive without the supplemental carbon source typically had larger patches of green cells in their cotyledons and/or hypocotyls. These patches of green cells were presumably capable of providing the seedling with photosynthate and other essential plastid-derived components necessary for survival until the first true green leaves could develop from the apical meristem. Similarly, *sco1-1 *seedlings that were able to survive when germinated in soil had large green patches (data not shown). Leaves and other photosynthetic tissues derived from the apical meristem in *sco1-1 *were green and visually indistinguishable from wild-type plants (Figure 3B).

To evaluate the structural development of chloroplasts in the cotyledons, we examined cells by light and transmission electron microscopy in 5-d-old wild-type and *sco1-1 *cotyledons. As expected, cotyledon mesophyll cells of light-grown wild-type seedlings contained numerous well-developed chloroplasts (Figure 3E). In contrast, chloroplasts were essentially absent from cotyledon mesophyll cells of typical *sco1-1 *seedlings except in the bundle sheath cells that surround the cotyledon vasculature (Figure 3F), consistent with the appearance of red autofluorescence (Figure 3D). In thin-sections from 'green-sectored' *sco1-1 *cotyledons (Figure 3H), we observed chloroplast-containing mesophyll cells directly adjacent to cells that are devoid of chloroplasts. The chloroplasts that developed in green *sco1-1 *cells appeared normal and showed characteristics of typical wild-type chloroplasts (Figure 3J).

Since chloroplast development was altered in *sco1-1 *seedlings, we stained seedlings for starch to determine if amyloplast development was also altered. Starch staining revealed that *sco1-1 *hypocotyls contained starch grains, indicative of the presence of amyloplasts in the endodermis (Figures 3K, L). Starch grains in *sco1-1 *root columella cells also appeared similar to those in wild type (data not shown). Consistent with the role of amyloplasts in gravity perception [13], gravitropism of hypocotyl, root, and inflorescence in *sco1-1 *was found to be similar to wild type (data not shown). These data indicate that amyloplast development is normal in the *sco1-1 *mutant seedlings.

### Transcript abundance in conditional-lethal *sco1 *alleles

**Figure 4:**
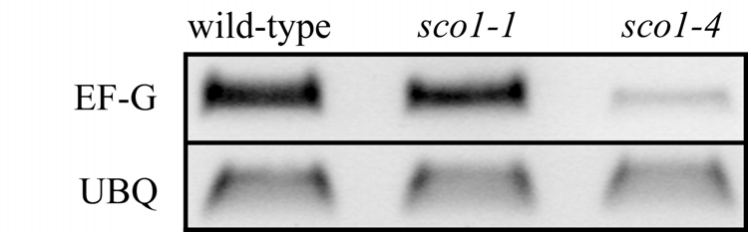
**Expression of SCO1**. RTPCR analysis of the EF-G transcript from wild-type, *sco1-1*, and *sco1-4 *seedlings demonstrates a lower abundance of transcript level in *sco1-4*. The TDNA insert in *sco1-4 *is located 14 base-pairs upstream of the ATG start site, directly affecting its transcription rate. The genetic lesion in *sco1-1 *does not appear to affect the level of *SCO1 *mRNA, as its level appears similar to that of wild-type. The level of the loading control *UBQ *transcript is similar between all three samples.

Given the location of the genetic lesions in *sco1-1 *and *sco1-4*, we wanted to determine if the relative abundance of *SCO1 *transcript in each mutant was related to the different albinism phenotypes of their cotyledons. Using primers specific to *SCO1*, we determined that *SCO1 *transcript abundance was similar in wild-type and *sco1-1 *seedlings (Figure 4). Since the EMS mutation in *sco1-1 *converts a glycine contained within the GTP-binding domain to an arginine, transcription was expected to be similar to wild-type. However, the level of transcript in *sco1-4 *is significantly reduced, which is consistent with the location of the T-DNA insert in the promoter region of *SCO1*. The level of transcript amplification of ubiquitin (*UBQ*) was similar in the mutants and wild type. We also found that SCO1 protein tagged with GFP was targeted to chloroplasts and to several non-photosynthetic plastids (data not shown), confirming data presented by Albrecht et al. [12].

### Embryo development in wild type and *sco1 *

**Figure 5:**
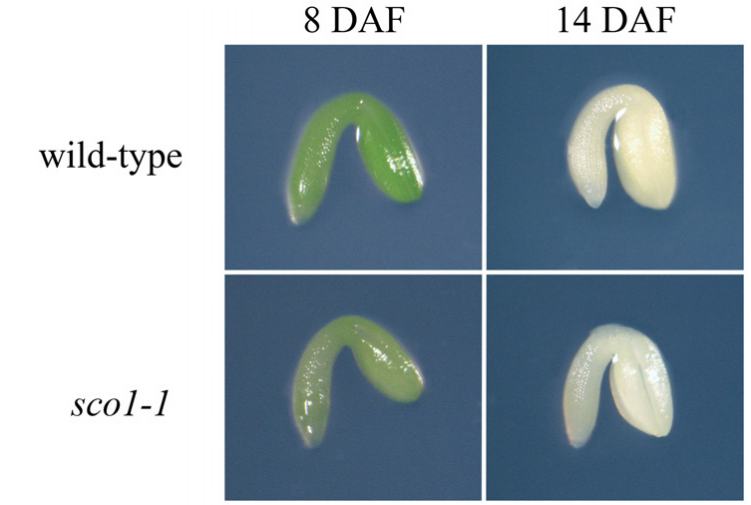
**Development of *sco1-1 *embryos**. Representative embryos were dissected from the middle of siliques on days 8 and 14 after anthesis. With respect to developmental rate and morphology, *sco1-1 *embryos were similar to wild type. The *sco1-1 *embryos were green from days 6 to 12, but appeared slightly paler compared to wild-type embryos of similar age. DAF = days old after fertilization.

Since Arabidopsis embryos are green during much of their growth, we examined embryos of *sco1-1 *plants to determine if chloroplast development was impaired in the mutant during embryogenesis. Embryos dissected from the middle of siliques between 5 and 15 days after fertilization (DAF) showed that *sco1-1 *and wild-type embryos were similar in both morphology and developmental rate (representative embryos from days 8 and 14 are shown in Figure 5). Embryos from both *sco1-1 *and wild type were observed to become visibly green around 6 DAF, and remained so until approximately 12 DAF when the chloroplasts began to dedifferentiate in preparation for dehydration and maturation of the seed. *sco1-1 *embryos dissected from siliques that were 11,12, or 13 DAF and the embryos were still green, were able to develop green hypocotyls and cotyledons when precociously germinated on agar growth medium, but embryos dissected after the embryos had turned white (>13 DAF) developed the characteristic white cotyledons seen in the *sco1-1 *mutant (data not shown). At 12 DAF, Arabidopsis embryos are in the early stages of desiccation and the onset of dormancy, and chloroplasts are beginning to dedifferentiate [11]. Since the *sco1-1 *phenotype could be rescued by bypassing the maturation stage of embryogenesis, the function of the mutant EF-G appears to be particularly critical during late stages of embryo development when eoplasts form.

### Expression levels of the Arabidopsis EF-Gs

**Figure 6:**
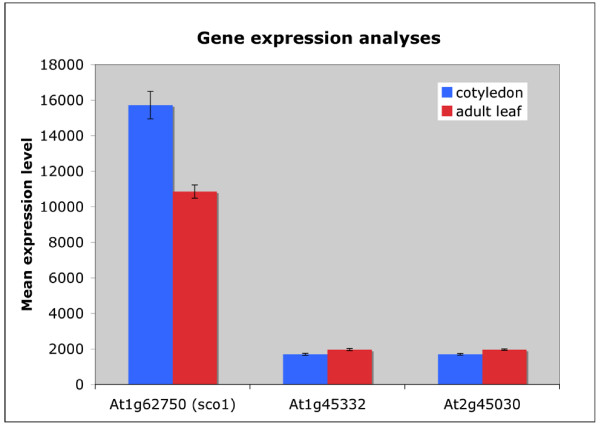
**Gene expression levels of the Arabidopsis nuclear-coded elongation factor Gs**. The Arabidopsis genome encodes for a total of three elongation factor Gs, including, *SCO1 *and two predicted mitochondrial-targeted EF-Gs. Transcript levels of *SCO1 *are highest in cotyledon tissue, with reduced levels in adult leaves. At1g45332 and At2g45030 transcript levels are highly reduced compared to *SCO1 *and remain constant between cotyledon and adult tissues. If these two EF-Gs are dual-targeted, they may be able to aid or compensate for the impaired activity of SCO1 in *sco1-1 *and *sco1-4*.

The most current annotation of the Arabidopsis genome predicts a total of three nuclear-encoded EF-Gs. Unlike the plastid-targeted *SCO1*, the two other EF-Gs (At1g45332 [GenBank:NM_103595] and At2g45030 [GenBank:NM_130067]) contain predicted mitochondrial-targeting sequences [14]. According to the subcellular prediction program TargetP, their targeting sequences may allow for dual targeting of the proteins to the mitochondria and plastids. During the course of Arabidopsis development, *SCO1 *is the most highly expressed of the EF-Gs, with expression levels peaking at 9.2 times the levels of At1g45332 and At2g45030 in cotyledon tissue (Figure 6; data compiled from Genevestigator [15]). Transcript levels of *SCO1 *are reduced in adult rosette tissue as compared to cotyledon tissue, whereas At1g45332 and At2g45030 mean expression levels remain relatively constant throughout development, but are always much lower than for *SCO1*.

## Discussion

### *sco1 *encodes for a translation elongation factor G

In general, the chloroplast genome encodes for genes that can be classified into several functional categories, including genes specific to transcription and translation within the plastid, photosynthetic genes, and genes involved in the synthesis of metabolic compounds [16]. Many of the components of the chloroplast proteome, however, are nuclear-encoded [17], including a number of factors that have been shown to be important in regulating translation of plastid genes [18,19]. For example, studies in various plant species indicated that protein initiation factors and elongation factors, including elongation factors EF-G and EF-Tu, are present in the nuclear genome and contain chloroplast-targeting sequences [20-25]. Much of our knowledge of plastid gene function in transcription and translation has drawn from structural and functional similarities to prokaryotic proteins that serve in a similar capacity. The presence of plastid-specific ribosomal proteins (PSRPs), however, indicates that at least some aspects of the translation mechanism in chloroplasts is unique to plants [26,27].

As shown here and by Albrecht et al. [12], the nuclear *SCO1 *gene encodes for a protein translation elongation factor G with a chloroplast-targeting signal sequence. Although biochemical activity has not been directly demonstrated for SCO1, similar functionality seems highly likely since the predicted amino acid sequence of SCO1 is over 50% identical to the *E. coli *EF-G *fusA *[GenBank:X00415], including conservation of glycine 132, which was changed to an arginine in the *sco1-1 *mutant.

During the elongation phase of plastid protein biosynthesis, the elongation factor EF-Tu binds to an aminoacyl-tRNA, which is then directed to the A site of the ribosome. The EF-G protein is then required to translocate the newly formed peptidyl-tRNA from the ribosomal A-site to the P-site. Although the *sco1-1 *mutant is viable when provided with sucrose during germination (Table 1), the two alleles we identified with T-DNA insertions in the *SCO1 *coding sequence (*sco1-2 *and *sco1-3*) have an embryo-lethal phenotype (Figure 2), indicating that *SCO1 *is an essential gene. The G to A base change in the *sco1-1 *mutant converted a conserved glycine residue to an arginine at position 132, which is between the P-loop and Switch I regions of the conserved GTP-binding domain found in the 70S-ribosome-binding region of elongation factors. Since the activity of EF-G is dependent upon hydrolysis of GTP [28], the amino acid change in *sco1-1 *may interfere with GTP hydrolysis activity in the mutant protein, affecting binding to or release from the ribosome. Since *sco1-1 *plants are viable once they produce leaves from the apical meristem, the mutant EF-G produced is likely to retain partial EF-G activity. However, we have not found reports describing a similar mutation in *E. coli*.

The *sco1-4 *allele, which contains a T-DNA insertion upstream of the *SCO1 *start site, most likely produces a normal EF-G but at reduced levels compared to wild type (Figure 4), which could cause the pale cotyledon phenotype (Figure 2D). The different cotyledon phenotypes of *sco1-1 *and *sco1-4 *is likely due to differences in the manner in which translation is altered. Previous research has demonstrated that in adult leaf tissues, proper plastid protein translation is absolutely essential for cell survival [29], and a similar reduction in protein translation in *sco1-1 *[12] reveals that plastid protein translation is also critical during seedling development.

### *sco1 *is critical during late embryogenesis and/or early germination

The plastid defects seen in *sco1-1 *and *sco1-4 *are most pronounced in seedling cells that were derived from the embryo. A few other published Arabidopsis mutants exhibit seedling-specific abnormal chloroplast development, including *white cotyledon 1 *(*wco1*) [30] and *sig*ma factor 6 (*sig6*) [31,32]. Mutations in *SIG6 *cause similar seedling stage-specific effects on chloroplast development to *sco1-1*, such as albino to pale-green cotyledons and normal leaf development. It was suggested that the *sig6 *mutant is able to produce normal chloroplasts in adult tissues due to redundancy in the role of sigma factors throughout development. In the *wco1 *mutant, the white cotyledon phenotype is highly dependent on light intensity, and the plants show various other defects in addition to the seedling albinism, including a marked reduction of chlorophyll content in adult rosette leaves. The *wco1 *phenotype is thought to result from a disruption of 16 S rRNA maturation, making it one of several mutants that appear to affect 16 S rRNA maturation and disrupt chloroplast development [33,34].

Since the eoplast to chloroplast transition is defective in *sco1-1 *and *sco1-4 *mutants, it appears that SCO1 activity is particularly critical during either the transition from chloroplast to eoplast, or when eoplasts redifferentiate into chloroplasts after germination. When we precociously germinated *sco1-1 *embryos before their chloroplasts had converted to eoplasts, green seedlings were obtained, indicating that some aspect of eoplast formation is critical for the *sco1-1 *mutant phenotype to develop. Because all plastid types would be expect to be impaired if eoplasts were abnormal, the presence of starch-containing amyloplasts in *sco1-1 *seedlings suggests that eoplast formation may be relatively normal in the mutant embryos and that the eoplast to chloroplast transition may be more demanding of EF-G activity than for the eoplast to amyloplast transition. Consistent with observations in Albrecht et al. [12], we found that the SCO1 tagged with GFP was targeted to chloroplasts. In addition to this previously determined chloroplast targeting, we found that the SCO1::GFP was also localized to non-photosynthetic plastids such as those in root and petal cells.

Even in the white *sco1-1 *cotyledons, chloroplasts were observed by chlorophyll fluorescence and microscopy in cells surrounding the vasculature (Figure 3D). In addition, while some *sco1-1 *seedlings appear almost entirely albino (Figure 3B), others show a variegated phenotype with sectors of 'wild-type' green cells (Figure 3G). When green sectors are present, they are mostly located around the cotyledon margin and the cells appear to contain a full complement of chloroplasts. It has been shown that lipid and starch deposition, which are associated with the progression of maturation in cotyledons of developing soybean embryos, begins in the interior cells of the organ and progresses to the periphery [35]. If maturation of Arabidopsis cotyledons follows a similar gradient, cells along the margin may not fully dedifferentiate their chloroplasts into eoplasts prior to seed maturation. Since we could rescue the *sco1-1 *phenotype by precocious germination, it is possible that the stages of plastid development that appear to be most dependent on SCO1 activity may be bypassed in a subset of cells that happen to arrest prior to full eoplast formation.

The lethality of the T-DNA insertion alleles (*sco1-2 *and *sco1-3*) is consistent with the hypothesis that SCO1 represents an essential gene in Arabidopsis involved in protein synthesis in plastids. There are at least two other predicted EF-Gs in the Arabidopsis genome, both of which are predicted to encode EF-Gs with mitochondrial-targeting and possibly plastid-targeting sequences. Dual targeting has been observed for other plant transcripts, including at least 17 of the Arabidopsis aminoacyl-tRNA synthetases [36]. At1g45332 and At2g45030 are over 98% identical to each other and show 43% identity and 62% similarity to SCO1, respectively, excluding the targeting sequences. If At1g45332 and/or At2g45030 are dual-targeted, it is possible that they may provide EF-G activity in at least some cell and/or plastid types that can aid, or compensate for, the impaired activity of SCO1 in the *sco1-1 *and *sco1-4 *mutants. It is also possible that one or both of these other EF-G genes can contribute to protein synthesis during later stages of plant development, which could allow the *sco1-1 *and *sco1-4 *mutants to develop green leaves. It is also possible that in the absence of normal SCO1 levels, expression of the other EF-Gs may be increased. However, expression analyses of these three EF-Gs in wild-type plants indicates that SCO1 expression greatly exceeds that of the other two EF-Gs in both cotyledons and mature leaves (Figure 6). More detailed analysis of expression levels and protein localization for all three EF-Gs during development will help distinguish between the various potential explanations. Given the lethal phenotype of T-DNA inserts in SCO1, however, neither of these other EF-Gs appears fully capable of providing sufficient EF-G function for plastid development in the absence of SCO1 activity during early stages of embryo development.

## Conclusion

The results presented here show that the EF-G encoded by the *SCO1 *gene in Arabidopsis is essential for plant growth since T-DNA insertions in the gene cause embryo lethality. The stage-specific phenotypes of the *sco1-1 *and *sco1-4 *mutants described here, and for the *wco1 *and *sig6 *mutants, reveal fundamental differences between plastid development in embryo-derived cells and cells derived from the apical meristem. Analysis of other seedling plastid-defective mutants should provide a better understanding of plastid formation during this critical period in plant development.

## Methods

### Plant material and growth conditions

The plants used for this study were of the Columbia ecotype of *Arabidopsis thaliana*. The *sco1-1 *mutant was isolated in a screen of 80,000 seedlings from 0.3% ethyl methanesulfonate (EMS, Sigma-Aldrich, Saint Louis, MO) mutagenized Arabidopsis M2 seeds as a seedling displaying white cotyledons but green meristematically-derived tissue. The *sco1-2 *(SALK_046154), *sco1-3 *(SALK_039084), and *sco1-4 *(Salk_025112) T-DNA insertion lines were obtained from the Arabidopsis SALK collection [37] at the Arabidopsis Biological Resource Center (The Ohio State University, Columbus, OH). The position of the T-DNA insert was confirmed through PCR amplification with the primer LBa-1 (located on the TDNA insert: 5'TGGTTCACGTAGTGGGCCATCG3') and primers flanking the predicted inserts (5'AAAAACAAAAGCAGACATCGAC3' for *sco1-2*, 5'GACCAAACAAAATCACAATAAG3' for *sco1-3*, and 5'ATGAAACACGAGCTATATTGAG3' for *sco1-4*).

Wild-type and all *sco1 *seed were sown on 1% agar growth medium containing 0.5-strength MS salts (Gibco/Life Technologies, Grand Island, NY) and 2% sucrose. The sown seeds were cold treated at 4°C for 48 h and then allowed to germinate and grow at 23°C in a growth room with a 12 h photoperiod under 60 to 70 μmol m^-2 ^s^-1 ^of light produced by a mixture of cool-white and warm-white fluorescent bulbs (General Electric, Louisville, KY). When the first true leaves had developed, seedlings were transferred to pots containing Scotts Plug mix (Scotts-Sierra, Marysville, OH). Plants were fertilized with K-Grow all purpose plant food (Kmart, Troy, MI) on a two week cycle.

### Identification and sequence analysis of *sco1*

An F2 mapping population was established between *sco1-1 *(Columbia ecotype) and the Landsberg *erecta *(LER) ecotype of Arabidopsis. The analysis of sequence polymorphisms in 450 F2 recombinant lines homozygous for *sco1-1 *placed the mutation in a 163 kilobase region on Chromosome 1, which was covered by bacterial artificial chromosomes (BACs) T3P18 and F23N19 [GenBank:AC007190]. We subcloned BAC F23N19 because it contained the bulk of the genetic interval. The pBeloBAC plasmid containing BAC F23N19 (Arabidopsis Biological Resource Center) was partially digested by Sau3AI (New England Biolabs, Beverly, MA) for 30 minutes at 37°C to obtain fragments approximately 10–15 kilobase in size. These fragments were ligated (T4 DNA ligase, New England Biolabs, Beverly, MA) at 15°C overnight to the *Bam*HI-digested (New England Biolabs, Beverly, MA) binary vector pCLD04541 (Arabidopsis Biological Resource Center). Plasmid DNA was introduced into *Escherichia coli *(DH5α) using a Gigapack III XL cosmid packaging kit (Stratagene, Cedar Creek, TX). *E. coli *colonies were screened using BAC F23N19 specific markers located every 10 kilobases to identify clones that provided coverage of the entire BAC. These were mated into *Agrobacterium tumefaciens *(GV3101) using the *E. coli *helper strain pRK2013. *sco1-1 *plants were transformed by floral dipping [38] and selected by growing seeds on 0.5-strength MS salts containing 1% agar and 50 μg/mL kanamycin (Sigma-Aldrich). Rescued plants were identified by their kanamycin-resistance and green cotyledons. Three candidate clones, defining an area no larger than 20 kb, were found to rescue the *sco1-1 *phenotype and were confirmed in the T2 generation. Sequencing of the seven genes in the identified interval revealed a mutation in a Tu family protein translation EF-G (At1g62750).

### Embryo dissection experiments

Using wild-type and *sco1-1 *plants that were similar in appearance, staged embryos were obtained from siliques formed from flowers that were dated upon reaching anthesis. Ovules were collected from the central portion of siliques that were from 5 to 15 d post anthesis (DPA) and the embryos were dissected from the ovules. Ovules of *sco1-2 *and *sco1-3 *were examined in 9-d-old siliques that were cut open and photographed using a Nikon SMZ1500 dissecting scope with a Nikon Digital Camera DXM1200 (Melville, NY).

### Microscopy

Plant materials were cut and placed into a 3% formaldehyde/gluteraldehyde solution in 0.1 M sodium cacodylate buffer, pH 7.4 (Electron Microscopy Sciences, Hatfield, PA) and fixed overnight at 4°C. The fixed samples were washed and post-fixed in 2% OsO_4 _at 4°C overnight. The samples were then washed, dehydrated, and embedded in spurs resin (Electron Microscopy Sciences, Hatfield, PA). For the cotyledon cross-sections, the embedded pieces were sectioned using an automated ultra-microtome and a glass knife. The sections were stained with bromophenol blue (Sigma-Aldrich, Saint Louis, MO) to reveal chloroplasts and cell walls. Images were captured using the brightfield function on a Nikon E800 microscope (Melville, NY). For the transmission electron microscope images, cotyledon pieces were sectioned using an automated ultra-microtome with a diamond knife (Pelco International, Redding, CA). The sections were stained with a 2% uranyl acetate solution and lead citrate as previously described [39]. Stained sections were observed and imaged using a JEOL-1010 Transmission Electron Microscope (JEOL USA, Inc., Peabody, MA).

### *sco1 *transcript analysis

RT-PCR was performed using the SuperScript III One-Step RT-PCR with Platinum Taq (Invitrogen Corp., Carlsbad, CA). A total of 50 nanograms starting RNA concentration were used in each reaction. *SCO1 *gene specific primers used for amplification were (For – 5'AAAAACAAAAGCAGACATCGAC3') and (Rev – 5'GGATCCTTAAGCAGCAACTTCTTGATCC3').

### *sco1 *localization

*SCO1 *was fused to a 35 S driven, C-terminal GFP construct utilizing the Gateway vector system (Invitrogen Corp., Carlsbad, CA). Gene specific primers with flanking *attB *sites were used to amplify the gene (5'GGGGACAAGTTTGTACAAAAAAGCAGGCTTCAACAATGGCGGCGGATGCTCTGAG3' and 5'GGGGACCACTTTGTACAAGAAAGCTGGGTCAGCAGCAACTTCTTCTTGATCCTTG3'). The expression clone was inserted into the donor vector pDONR201, and subsequently transformed into One Shot TOP10 chemically competent cells (Invitrogen Corp., Carlsbad, CA). Transformed clones were identified on Luria-Bertani (LB) medium containing 50 μg/ml kanamycin and tested with gene specific primers. A miniprep purification (Qiagen, Valencia CA) was then done on a positive clone and used in a recombination reaction with the destination vector pVRGFP (provided by Vincente Rubio and Xing Wang Deng). The ligated plasmid was subsequently transformed into One Shot TOP10 chemically competent cells. Transformed clones were identified on LB medium containing 50 μg/ml spectinomycin and tested with gene specific primers. A positive clone was mated into *Agrobacterium tumefaciens *(GV3101) using the *E. coli *helper strain pRK2013 and used to transform wild-type Arabidopsis plants (ecotype Columbia) by floral dipping [38]. Rescued plants were identified by their gentamycin-resistance (200 μg/ml) and green cotyledons.

### Sequence alignments

The publicly available program TCOFFEE was used to produce sequence alignments of *sco1*, At1g45332, At2g45030, and the *E. coli *EF-G (*fusA*). Outputs were designed by BOXSHADE [40].

## Authors' contributions

NJR participated in the design of the study, carried out all experiments, and drafted the manuscript. RPH conceived of the study, participated in its design and coordination, and assisted with manuscript preparation.
